# MR-CT multi-atlas registration guided by fully automated brain structure segmentation with CNNs

**DOI:** 10.1007/s11548-022-02786-x

**Published:** 2022-11-05

**Authors:** Sina Walluscheck, Luca Canalini, Hannah Strohm, Susanne Diekmann, Jan Klein, Stefan Heldmann

**Affiliations:** Fraunhofer Institute for Digital Medicine MEVIS, Bremen, Germany

**Keywords:** CT, Registration, CNN, Deep learning

## Abstract

****Purpose**:**

Computed tomography (CT) is widely used to identify anomalies in brain tissues because their localization is important for diagnosis and therapy planning. Due to the insufficient soft tissue contrast of CT, the division of the brain into anatomical meaningful regions is challenging and is commonly done with magnetic resonance imaging (MRI).

****Methods**:**

We propose a multi-atlas registration approach to propagate anatomical information from a standard MRI brain atlas to CT scans. This translation will enable a detailed automated reporting of brain CT exams. We utilize masks of the lateral ventricles and the brain volume of CT images as adjuvant input to guide the registration process. Besides using manual annotations to test the registration in a first step, we then verify that convolutional neural networks (CNNs) are a reliable solution for automatically segmenting structures to enhance the registration process.

****Results**:**

The registration method obtains mean Dice values of 0.92 and 0.99 in brain ventricles and parenchyma on 22 healthy test cases when using manually segmented structures as guidance. When guiding with automatically segmented structures, the mean Dice values are 0.87 and 0.98, respectively.

****Conclusion**:**

Our registration approach is a fully automated solution to register MRI atlas images to CT scans and thus obtain detailed anatomical information. The proposed CNN segmentation method can be used to obtain masks of ventricles and brain volume which guide the registration.

## Introduction

Computed tomography (CT) imaging of the brain is widely used in radiology as it provides good image contrast to identify hemorrhages, cerebrovascular lesions and tumors. To determine the best treatment, each pathology has to be detected and localized as precisely as possible. CT is the modality of choice in acute patient care and is provided 24/7/365 in many hospitals. An emerging shortage of radiologists could be outweighed by precise automated CT exam reporting. The diagnosis and subsequent therapy depends on the anatomical localization, as symptoms and neurological disorders correspond with the affected brain area. Due to the poor soft tissue contrast of CT, precisely determining anatomical structures and differentiating brain areas is challenging. Magnetic resonance imaging (MRI) is used to highlight specific structures thanks to higher soft tissue contrast and the possibility of acquiring different MRI protocols. MRI mainly is the modality of neuroscience and elective clinical work-up. In daily practice, there is limited availability and there are controversies concerning the feasibility in acute symptomatic patients. Even with MRI, a precise, individual segmentation is time consuming and not feasible in clinical routines.

A possible solution to obtain masks of anatomical structures is to use an already existing brain atlas. In atlas-based approaches, a template intensity image is registered to the target image, and the resulting deformation is then applied to the anatomical labels of the template to match the target space. In this way, the existing information of the atlas image can be transferred to the image of interest [1]. To better address the variability between scans of different subjects, multi-atlas registration can be used [2]. With this approach, the target image is registered to multiple atlas images and the resulting label images are combined via majority voting algorithms [2–4].

There are multiple MR-based anatomical atlases available to transfer different labeled regions to new unlabeled images. To the best of our knowledge, there is no detailed CT-based anatomical brain atlas, and few registration-based approaches have been proposed for transferring anatomical information from an MR image to a CT scan. This is a challenging task and requires reliable multi-modal, inter-subject, non-rigid CT-MR registration. In the approaches proposed by [5–7], the task is reduced to a mono-modal registration problem by decomposing it into two steps. First, CT images are synthesized into MRI images, and then the registration is performed between the synthesized MRI images and the MRI atlas.

Moreover, in [8] the authors propose a method to create an average CT atlas. Therefore, they first create an average CT image and then register multiple MRI atlas images to the average image. Finally, the anatomical labels from the MRI atlases are fused completing the final average CT atlas.

Furthermore, the authors in [9] investigate an atlas-based method to segment ventricles in CT images. However, when it comes to CT images, it would be beneficial to delineate a larger number of anatomical structures. The authors in [10] propose a registration-based method to build a CT head atlas with anatomical structures for the Chinese population. However, they manually correct the segmentation and do not use MR atlas images. Direct multi-modal registration usually is eased by utilizing additional information and correspondence structures in the distance measure computation. Gao et al. [11] extract the midsagittal plane and use brain surface matching. Chen et al. [12] propose a combination of landmarks and mutual information (MI) as similarity measure to include local and global anatomical structure. Learning-based approaches, as described in [4], aim to overcome the disadvantages of multi-modal similarity measures such as MI. To evaluate the performance of the label propagation on MRI scans, Dubost et al. [13] introduce the computation of the overlap from automatically segmented ventricles and choose the result with the highest Dice score.

In this work, we present a novel enhanced multi-modal, multi-atlas registration approach to propagate anatomical labels from an MRI atlas to new unseen CT brain scans. Our approach builds on deformable registration utilizing corresponding structures (brain parenchyma and ventricles) as extra input to guide the registration process between the CT image and the MR atlas. On that account, we propose a convolutional neural network solution to automatically segment brain volume and ventricle system in CT images.

## Methods and material

**Fig. 1:**
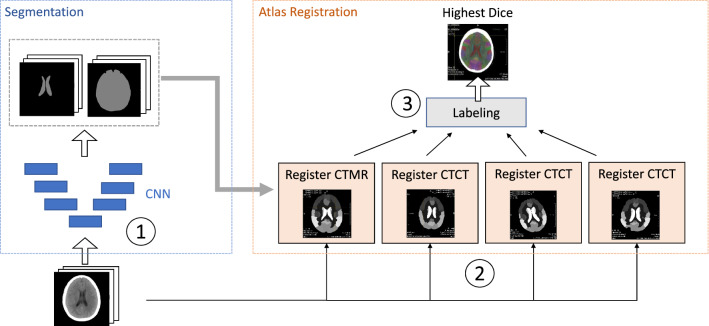
Overview of our atlas registration approach: Step (1): automated segmentation of brain structures with CNNs, (2): multi-atlas registration, (3): create label image

Our solution is divided into three steps. Firstly, brain volume and ventricles of each CT scan are automatically segmented by convolutional neural network (CNN) approaches. In the second step, we perform multi-atlas registration using the segmentation masks as guidance structures for the registration. We assume that a better alignment of the ventricles also leads to more precise propagation of all anatomical labels. Details are described in Section “Atlas Registration.” In our multi-atlas solution, CT scans are registered to an MRI brain atlas and with a mono-modal approach to three different pre-computed CT atlas images, such that we obtain four label images for each CT input. Finally, in the third step, we choose the label image with the highest ventricles Dice values. An overview of our approach is shown in Fig.  1. Registering a CT to all of the four atlas images and obtaining the final label image takes around 90 seconds on a system with NVIDIA GeForce RTX 2070 Super.

### Data

Our experiments are based on the publicly available data set provided by the Radiological Society of North America (RSNA) in collaboration with members of the American Society of Neuroradiology and MD.ai in the context of the RSNA challenge for intracranial hemorrhage detection [14]. The data set includes over 25,000 CT slices of the head, labeled with the type of hemorrhage, if present. We reconstructed 3D volumes from the 2D CT scans and selected a subset of 220 “normal” 3D volumes without hemorrhage. This corresponds to 220 subjects with one scan per subject. For this data set, ventricles (right and left lateral and 4th) and brain volumes were manually segmented by three radiologists with 3 months, 6, and 12 years of experience annotating CT images and using SATORI software [15]. All radiologists were trained by an experienced neuroradiologist, data sets were randomly distributed among radiologists, and each data set was segmented by only one radiologist. For final homogeneous segmentation, all data sets were reviewed by an experienced neuroradiologist. Additionally, we randomly selected 10 abnormal CT volumes with hemorrhages to test our method on disease cases. For the atlas registration, we use the AAL1 MRI Atlas with added ventricle labels [16].

### Segmentation

We propose an automatic method to segment brain ventricles and parenchyma and use it as guidance to register new CT scans, for which no ground truth is available. CNN solutions already demonstrated to be a valid alternative for CT segmentation [17]. For this task, we utilize the “no new U-Net” (nnU-Net) deep learning method [18] that showed to achieve state of the art results in several medical imaging segmentation tasks. It is a self-configuring framework that automatically adapts to the data set used for training. An advantage of the nnU-Net approach is the automatic preprocessing depending on the type of training data used. As described in [18], the training data are globally clipped to the intensity range of the 0.5 to 99.5 percentile and z-score normalization is performed based on mean and standard deviation. For what concerns the architecture, the input patches size is automatically set to 20 $\times$ 376 $\times$ 376, with a batch size equal to 2. The 3D U-Net has a 5 levels depth, with LeakyRelu and batch normalization applied after every convolution operation. During training, the data are augmented by random rotation and scaling, additive brightness augmentation, gamma scaling and rigid transformation. Moreover, the loss function is composed of the sum of cross-entropy and Dice loss. The networks are trained for 1000 epochs, with an epoch defined on 250 mini-batches.

In this work, two distinct nnU-Net models have been trained to segment the ventricles, and the brain volume as this led to the best results. In both models, the training and validation set includes 198 cases, whereas a disjoint test set of 22 cases is available to test the trained model. The test and training sets are subsets of the previously described 220 normal scans with manual segmentation (Section “Data”).

### Atlas registration

As the robust multi-modal, inter-subject, non-rigid registration of medical images is an extremely challenging task, we incorporate multiple structure and landmark guidance into our solution. In our method, we combine mono-modal with multi-modal atlas registration. We assumed that the creation of CT atlas images through CT-MR registration could be beneficial over the mere use of multi-modal registration, as a mono-modal approach is known to be less prone to errors, especially for inter-patient scenarios as discussed here. However, our starting point is an MRI atlas that consists of an intensity image and corresponding labels, such that $\text {MR}(x)$ is the intensity and $\text {MR}^\text {Label}(x)$ is the anatomical label at position *x*. Then, we use multi-modal registration to propagate the labels to CT. To this end, we register the intensity images and subsequently warp the labels from MR to CT. That is, we compute a deformation vector field *y* such that $\text {CT}(x)\approx \text {MR}(y(x))$ and we define $\text {CT}^\text {Label}(x):= \text {MR}^\text {Label}(y(x))$.

#### Registration approach

We use a variational registration scheme that builds on normalized gradient fields (NGF) image similarity measure, second-order curvature and volume regularization of the deformation vector field. NGF has been proven to be a reliable distance measure in multi-modal CT-MR [19] as well as mono-modal CT-CT registration scenarios [20]. Furthermore, to improve robustness and accuracy we incorporate additional knowledge by adding penalty terms that enforce the alignment of the corresponding masks for brain and ventricles and centers of gravity (COG) of the ventricles, similar to [21, 22]. The COG could lie outside of the ventricle volume, but we are not searching for an anatomical meaningful landmark but rather a sensible reference point that can be extracted out of the ground truth that we have right now.

To be specific, in our setting the CT image is the so-called fixed or reference image *R* and the MR is the so-called moving or template image *T* that shall be aligned on a domain $\Omega \subset \mathbb {R}^3$ modeling the field-of-view of *R*. Furthermore, we assume corresponding segmentations for brain parenchyma (BP), left and right lateral ventricle (LLV, RLV) and fourth ventricle (FV), that are given as binary masks $M^R_\ell , M^T_\ell$ for $\ell =\text {BP}, \text {LLV},\text {RLV},\text {FV}$. Moreover, we consider combined masks for all ventricles, i.e., we set $M^R_\text {V} := \sum _{\ell \in {\mathcal {V}}}M^R_\ell$ for ventricle labels ${\mathcal {V}}:=\{\text {LLV}, \text {RLV}, \text {FV}\}$ and $M_\text {V}^T$ accordingly. Additionally let $r_\ell ,t_\ell \in \mathbb {R}^3$, $\ell \in {\mathcal {V}}$ be the centers of gravity (COGs) of the different ventricles, i.e., $r_\text {LLV}$ is the COG of $M^R_\text {LLV}$, $t_\text {LLV}$ is the COG of $M^T_\text {LLV}$, etc.

For the registration, we then minimize the following objective function w.r.t. to deformation vector field *y*:

1 $$\begin{aligned} \begin{aligned} J(R, T(y))&= \text {NGF}(R, T(y)) + \frac{\alpha }{2} \sum _{k=1}^3\Vert \Delta y_k\Vert _{L^2(\Omega )}^2 \\&\quad + \beta \int _{\Omega }^{}\psi (\det \nabla y(x))dx \\&\quad + \frac{\gamma }{2} \Big (\Vert M^T_\text {BP}(y)-M^R_\text {BP}\Vert ^2_{L^2(\Omega )} \\&\quad + \Vert M^T_\text {V}(y)-M^R_\text {V}\Vert ^2_{L^2(\Omega )}\Big ) \\&\quad + \frac{\delta }{2}\sum _{\ell \in {\mathcal {V}}} \Vert y(r_\ell )-t_\ell \Vert _2^2 \end{aligned} \end{aligned}$$

with weights $\alpha , \beta , \gamma , \delta > 0$, NGF distance measure

2 $$\begin{aligned} \text {NGF}(R,T) = \frac{1}{2} \int _{\Omega }^{} 1 - \left( \frac{ \left\langle \nabla R(x),\nabla T(x)\right\rangle _{\varepsilon _R \varepsilon _T} }{ \Vert \nabla T(x)\Vert _{\varepsilon _T} \, \Vert \nabla R(x)\Vert _{\varepsilon _R} } \right) ^2 dx \end{aligned}$$

where $\left\langle x,y\right\rangle _{\varepsilon } := x^\top y+\varepsilon$, $\Vert x\Vert _\varepsilon := \sqrt{\langle x,x\rangle _{\varepsilon ^2}}$ and $\varepsilon _R,\varepsilon _T>0$ are the so-called edge-parameters controlling influence of noise in the images. The weights are fixed and were determined empirically. In addition to penalizing the second-order (Laplacian) derivatives by the so-called curvature regularization, we add an additional term penalizing the Jacobians of the deformation, respectively, volume changes with the function $\psi (t)=(t-1)^2/t$ for $t>0$ and $\psi (t):=\infty$ for $t\le 0$. Note that $\psi (1)=0$ and $\psi (t)=\psi (1/t)$ and thus volume growth or shrinkage are penalized symmetrically, and $\psi (t)=\infty$ for $\det \nabla y \le 0$ prevents local changes in the topology and thus unwanted mesh folds.

The optimization is done by using a multi-level approach with L-BFGS.

#### Multi-modal atlas registration

In general, our approach builds on a single MR atlas that is transferred to CT as described before. However, to achieve better performance and coverage of anatomical variations, we bootstrap the MR atlas to a multi-modal MR-CT multi-atlas. To this end, all CT images in our data set (220 cases) were registered with the MR atlas intensity image and labels were propagated from MR to CT, so that we obtained a label image $\text {CT}^\text {Label}$ for each CT scan. Afterward, we manually selected three CT images along with the propagated label images that had the highest ventricular Dice values ($\ge$94%), so that our multi-modal multi-atlas consisted of one MR and three CT atlases. The number of chosen CT images could easily be adapted to incorporate more variability. We are aware that typical atlas based approaches consist of a much larger number of atlases [23].

However, we limit ourselves to three images because first, the total number of atlases should be balanced with the size of our data set. Using 10-20 images would mean that we are actually using 5–10% of the data set as atlases. While this might lead to a larger anatomical variety and thus better registration results, it would still bias the validity of the evaluation of our methodology. Second, we describe a bootstrap strategy to improve atlas registration using labels from a single MR atlas. The accuracy of bootstrapped CT labels is therefore highly dependent on the initial CT-MR registration quality, limiting the set of possible CT atlas candidates to those with very good CT registration quality. For this reason, we decided to use only the smallest possible number of three CT images for the evaluation of our approach, which is about 1.5% of the data.

The multi-atlas registration for a new unseen CT image works as follows. We use the approach from Section “Registration approach” to independently register a new CT image to each of the four atlas images, such that we obtain four registration results. We use the Dice overlap of the ventricles as a quality criteria, as these labels are available for all atlas images as well as the new unseen CT image by our automatic CNN segmentation described in Section “Segmentation.” Thus, we are globally choosing the warped anatomical label image of the atlas with the highest ventricle Dice. We are aware that in multi-atlas scenarios it is common to use a local label fusion approach, namely majority voting [2–4]. We implemented this during development, but then focused on the previously described global Dice approach.

**Table 1:** Segmentation results: Mean Dice coefficient and mean Hausdorff Distance (in mm), with related standard deviation. The test set consists of 22 volumes

**Metric**	LLV	RLV	4thV	Brain volume
Dice	$0.89\pm 0.04$	$0.89\pm 0.03$	$0.89\pm 0.06$	$0.97\pm 0.01$
Hausdorff	$15.61\pm 18.08$	$11.60\pm 21.39$	$34.65\pm 59.55$	$15.30\pm 3.04$

**Fig. 2:**
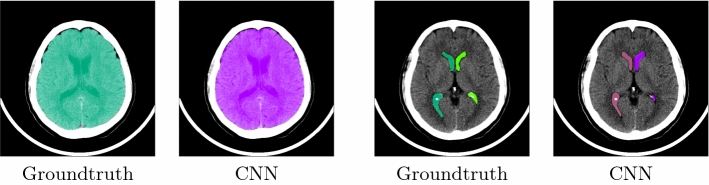
Automatic segmentation results for brain volume (left) and ventricles (right). The first and third images show the ground truth masks, whereas the second and fourth display the automatically generated masks

**Fig. 3:**
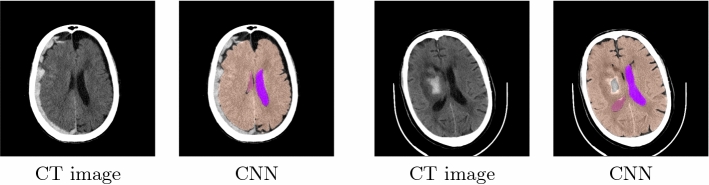
Disease cases: Automatic segmentation results for brain volume and ventricles. The brain volume (in orange) and the left and right later ventricles (in pink and purple). The pathological is visible in the right hemispheres of the slices

## Results

To evaluate both the segmentation and registration solutions, we compute the Dice coefficient for the ventricles and the brain volume (mean and std) as well as the Hausdorff distance (in mm) for the two structures.

### Segmentation

The segmentation task was tested on a data set of 22 volumes that were excluded from the CNN training. The corresponding results are presented in Table 1. The segmentation method for the brain volume achieves the highest Dice coefficient of 0.97, whereas the segmentation of the ventricles leads to a Dice of 0.89. The high Hausdorff values are especially due to the fact that parts of other nearby structures are wrongly assigned. In particular, for the very small 4th ventricle, parts of the LLV and RLV are misidentified as part of the 4th ventricle. Figure 2 shows exemplary results for a test case. Moreover, Fig. 3 displays the qualitative results on two cases with disease, in which the brain volume and the ventricles are automatically segmented.

**Table 2:** Registration results with manual and automatic segmentation for the test data set with 22 cases: Dice coefficient, Hausdorff distance (HD), 95% Hausdorff distance (HD95, =95% quantile of surface distances) and average surface distance (AVD) in mm with mean ± standard deviation, respectively

	Ventricles		Brain volume	
Metric	Manual	CNN	Manual	CNN
Dice	$0.92\pm 0.02$	$0.87\pm 0.03$	$0.99\pm 0.01$	$0.98\pm 0.01$
HD	$15.03\pm 8.66$	$16.12\pm 7.80$	$8.68\pm 4.24$	$11.76\pm 3.41$
HD95	$2.72 \pm 3.21$	$3.89 \pm 2.55$	$0.54 \pm 0.24$	$3.01 \pm 1.30$
AVD	$0.28\pm 0.21$	$0.42 \pm 0.21$	$0.07\pm 0.05$	$0.41\pm 0.25$

**Fig. 4:**
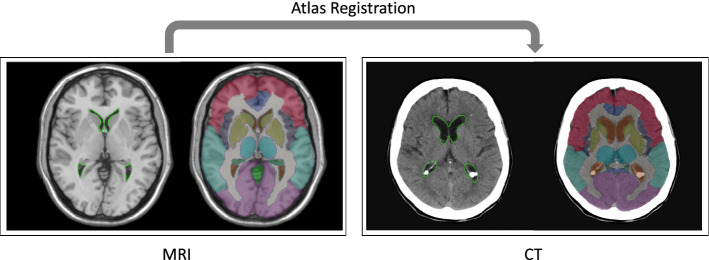
Exemplary registration result: Overlay of only the ventricle label and all anatomical labels on MRI atlas (left) and CT volume (right)

### Registration

For the registration task, we use 22 test CT scans with (A) ground truth segmentation masks, (B) automatically segmented ventricles and brain volume by the CNN. By using set (A), we evaluate the registration performance without considering the automatic segmentation results, as we register with guidance of the ground truth segmentation masks. To test the entire proposed pipeline, we then used the test set, where the guidance masks were generated by the CNN. The results are presented in Table 2. Qualitative results for the registration are shown in Fig. 4.

**Fig. 5:**
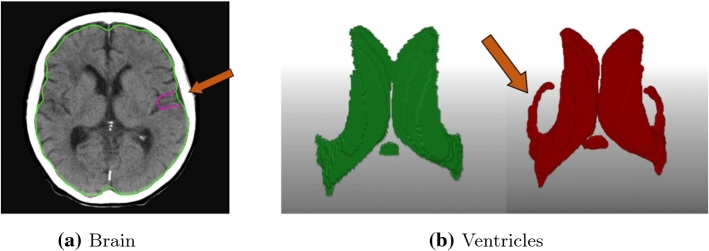
Visualization of cases with high Hausdorff distance for **a** brain volume and **b** ventricles with ground truth in red/pink and atlas result in green. The arrows indicate the area with differences

It is noticeable that the Hausdorff distance (HD) for both manual and automatic CNN-based segmentation is quite large compared to the good Dice values. This is because the segmentations have different levels of detail. In the ground truth masks, some sulci or fissures are precisely segmented with high detail and are not part of the brain volume. In contrast, the atlas brain mask is segmented at a coarser level and does not contain such details. Therefore, the sulci cannot be accurately mapped by registration, resulting in larger Hausdorff values. An example is shown in Fig. 5a. Similarly, the distances for the ventricles are large when the subhorn of the lateral ventricles is well segmented in the ground truth, which is not the case in the atlas. Such a case is shown in Fig. 5b. It is well known that the Hausdorff distance is very sensitive to such outliers. For this purpose, we also provide more robust 95% Hausdorff distance (HD95) and average surface distance (AVD) [24, 25], confirming the good Dice values, see Table 2.

#### Robustness

**Fig. 6:**
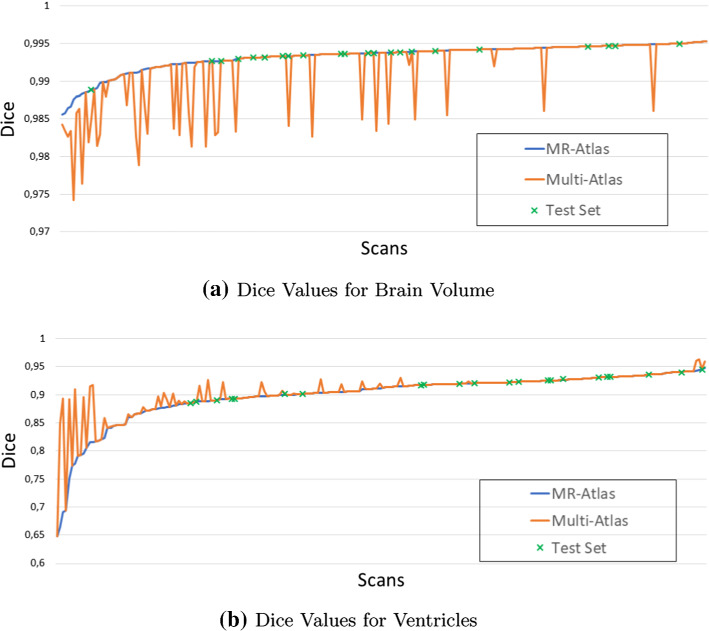
Dice values for ventricles and brain volume when using only the MR atlas or the multi-atlas. The data set consists of all of the 217 CT scans

We claim that adding CT images as auxiliary atlas images makes our overall approach more robust. To evaluate that, we compared the performance on the whole data set when using only the MR atlas versus using the multi-atlas with three CT images. The results are shown graphically in Fig. 6. We observed that the ventricle Dice is significantly improved for multiple cases when using the multi-atlas.

In addition to using a multi-atlas, we also utilize structure guidance to improve the registration performance. Table 3 shows a comparison of the registration metrics when using no guidance, only mask alignment and the full proposed method with additional landmark alignment. We conduct this experiment on our test data set of 22 CT scans with manual segmentation masks. The ventricle Dice of the registration without guidance increased from 0.72 to 0.92 when using landmark and mask alignment.We also applied the Wilcoxon test for dependent samples. The difference between not using any guidance and guiding with masks is significant with p-values for Dice and Hausdorff distance of $10^{-5}$ and $7\times 10^{-4}$, respectively. The use of additional landmark guidance does not lead to a significant improvement over using only the masks (p-value of 0.061).

**Table 3:** Registration results without guidance vs guided by masks or masks and landmarks: Mean Dice coefficient and mean Hausdorff Distance (in mm), with related standard deviation. Data set includes 22 manually segmented test scans

		Guidance by		
	Metric	None	Masks	Masks & landmarks
Ventricles	Dice	$0.72\pm 0.05$	$0.91\pm 0.02$	$0.92\pm 0.02$
	Hausdorff	$20.37\pm 7.11$	$16.25\pm 7.87$	$15.03\pm 8.66$
Brain Volumes	Dice	$0.94\pm 0.02$	$0.99\pm 0.01$	$0.993\pm 0.01$
	Hausdorff	$15.36\pm 3.40$	$8.74\pm 4.23$	$8.68\pm 4.24$

#### Scans with diseases

As mentioned earlier, our method was developed with normal CT images only and our training data did not include CT scans with pathologies. Therefore, we obviously cannot expect the same performance as with healthy data. Nevertheless, we tested our approach on a few selected CT scans with pathologies to get a first impression of the behavior on scans with diseases.

**Fig. 7:**
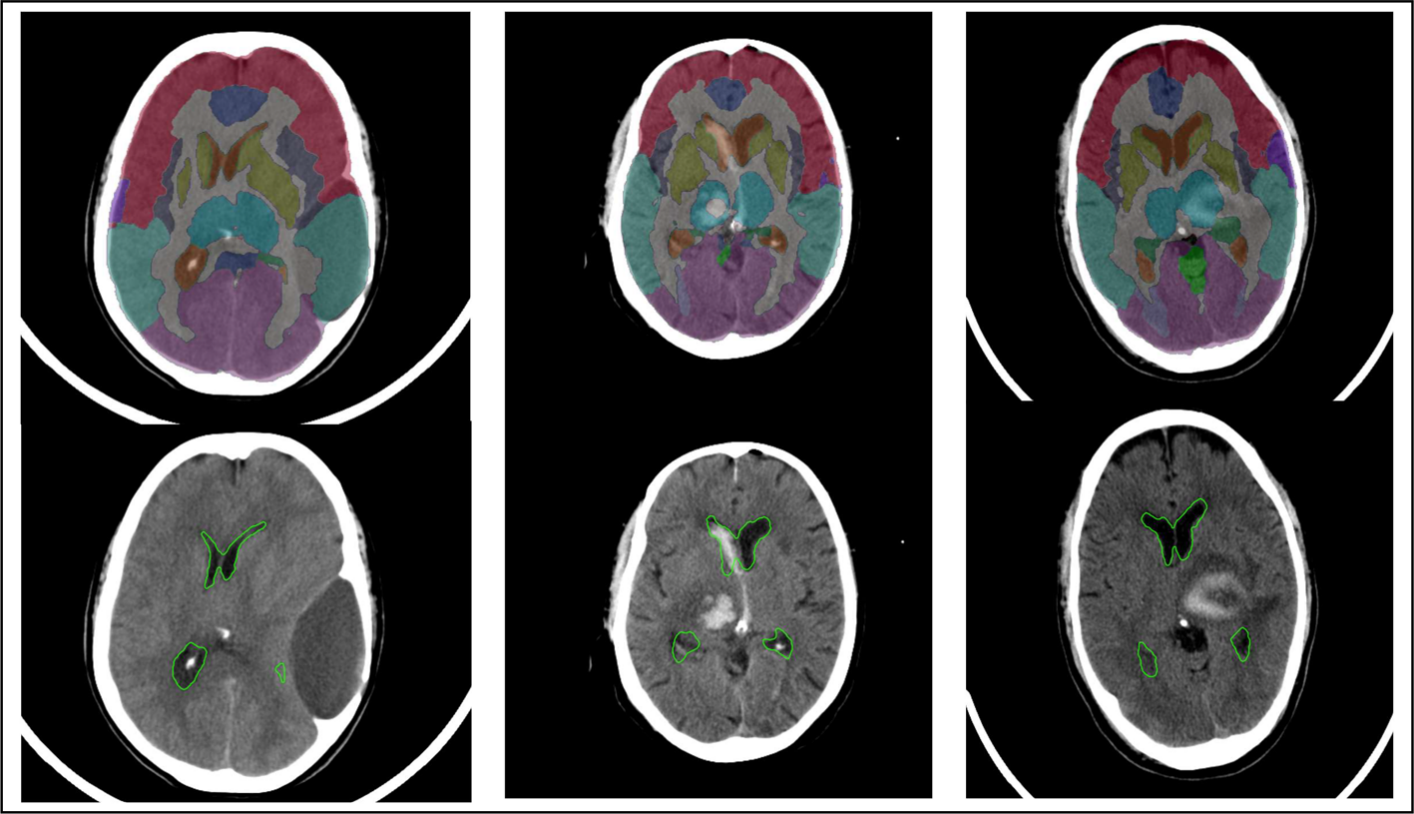
Three exemplary CT scans with pathologies and atlas labels that were propagated with our approach. First row: all atlas labels, second row: only label for lateral ventricles

Three examples are shown in Fig. 7. Quantitative evaluation is not provided as ground truth segmentation masks for our data are not available at this time and expert feedback is expected in the future.

## Discussion

We presented a CNN segmentation guided multi atlas registration method showing reasonable results and demonstrating robustness and accuracy of our approach.

The segmentation method achieves good results in automatically delineating brain volumes and lateral ventricles in healthy patients. In particular, qualitative results on volumes with diseases show that the method achieves a good delineation of the structures even if the lateral ventricles are compressed in the right hemispheres. Moreover, the brain volume is also well segmented, as the pathological area is not included in the automatically segmented brain volumes (see Fig. 3).

The proposed multi-atlas registration also shows robust and accurate performance in our experiments. Clearly, the registration outcome depends on the accuracy of the used guidance segmentation. However, we have shown that registration works well with both ground truth and automatically generated masks with slightly superior numerical results with the manual segmentation (cf. results in Table 2). Our evaluation is limited by the relatively small test set (22 cases), and evaluation on a larger data set is still ongoing. Admittedly, the main limitation of our evaluation is the missing ground truth for other anatomical structures than the ventricles and brain volume. We have to evaluate our method on the same structures that are used for guiding the registration which can be seen as a bias. However, we showed the registration results to radiology experts and got very positive feedback as some anatomical structures can be identified with our method that are very hard to segment on CT for humans. We hope to obtain masks for some structures that can reliably be segmented by experts in the future, to evaluate for example the results for the thalamus label. So far, assuring the accurate and consistent segmentation of such structures exceeded our capacities.

Furthermore, we found in our experiments that leveraging the single MR atlas with a bootstrapped multi-CT-MR atlas generally leads to much more robust and accurate results. However, instead of selecting CT images with the highest Dice values for our combined MR-CT multi-atlas, other criteria such as anatomical variability could be considered.

## Conclusion

In this paper, we presented a novel multi-atlas registration approach to obtain anatomical labels on CT scans using a standard MRI brain atlas. By using the detailed MRI information, we overcome the problem of creating an anatomical CT atlas. Furthermore, synthesizing MR images from CT, as found in the literature, is not needed as we directly use an MR atlas. Our method combines multi- and mono-modal registration and incorporates structure guidance with automatically segmented brain structures with CNNs. Thus, our registration guidance requires no manual interaction.

As future work, further improving the CNN segmentation to simultaneously segment several brain structures will be investigated. Moreover, we plan to further validate our approach also on pathological brain scans.
